# Implementation of a Laboratory-Developed Test for the Diagnosis of *Mycoplasma pneumoniae* Using a High-Throughput Approach

**DOI:** 10.3390/pathogens14070692

**Published:** 2025-07-14

**Authors:** Valeria Conciatori, Sarah Di Sopra, Elisa Franchin, Ioannis Bekas, Giuseppe Di Pietra, Ignazio Castagliuolo, Cristiano Salata, Claudia Del Vecchio

**Affiliations:** 1 Department of Molecular Medicine, University of Padua, 35121 Padua, Italy; valeria.conciatori@studenti.unipd.it (V.C.); sarah.disopra@studenti.unipd.it (S.D.S.); elisa.franchin@unipd.it (E.F.); ioannis.bekas.1@studenti.unipd.it (I.B.); ignazio.castagliuolo@unipd.it (I.C.); 2 Microbiology and Virology Diagnostic Unit, Padua University Hospital, 35121 Padua, Italy; 3 UOC Medicina di Laboratorio, Ospedale San Bassano, ULSS 7 Pedemontana, Bassano del Grappa, 36061 Vicenza, Italy; giuseppe.dipietra@aulss7.veneto.it

**Keywords:** *Mycoplasma pneumoniae*, Panther Fusion^®^ system, Quant Studio™5, respiratory infections, molecular diagnostics

## Abstract

*Mycoplasma pneumoniae* is a significant causative agent of atypical pneumonia in both children and adults. Timely and accurate diagnosis is crucial for appropriate patient management. Conventional methods for detecting *M. pneumoniae*, such as culture and serology, exhibit several limitations regarding sensitivity, specificity, and turnaround time. In contrast, real-time PCR is considered the most reliable, rapid, and sensitive technique for the diagnosis of *M. pneumoniae* infection. In this study, we adapted and validated an in-house real-time PCR assay for use on the fully automated Panther Fusion^®^ System. The validation process included two artificial samples, five external quality controls, and sixty-two patient samples. We evaluated the performance in terms of precision, sensitivity, linearity, and analytical sensitivity, comparing it to the original in-house assay. The Panther Fusion^®^ System demonstrated a broad dynamic range (16–1.6 × 10^7^ copies/reaction), a robust correlation (94%) with the in-house assay, and comparable sensitivity (46 copies/mL vs. 25 copies/mL). The concordance between the in-house real-time PCR and the Panther Fusion^®^ System was 100% for both clinical samples and external quality controls. The adaptation of the test to the Panther Fusion^®^ System enabled the inclusion of *M. pneumoniae* among the pathogens monitored for respiratory infection surveillance. Throughout 2024, we analyzed 2567 samples, with a peak positivity rate of 38% observed in August. These findings underscore the significance of employing the *M. pneumoniae* diagnostic assay on the Panther Fusion^®^ System which proves valuable for the detection of *M. pneumoniae* infections. This platform offers the advantages of increased automation and greater throughput potential compared to other platforms, enhancing the efficiency of respiratory pathogen detection in clinical settings.

## 1. Introduction

*Mycoplasma pneumoniae* is a very small, cell wall-lacking intracellular bacterium, first isolated in 1944 [1]. *M. pneumoniae* is a human-restricted pathogen capable of causing a wide spectrum of respiratory diseases, from mild upper respiratory tract infections to severe community-acquired pneumonia (CAP) and extrapulmonary complications in up to 40% of children and 20% of adults [2,3].

*M. pneumoniae* is endemic worldwide and circulates year-round, with peaks in the summer and autumn seasons and epidemic outbreaks every 4 to 7 years [3,4]. The infection spreads through respiratory droplets and has a relatively long incubation period (1–3 weeks). The most susceptible populations are children over 5 years of age and young adults [5]. At present, no approved vaccine is available [1].

Common symptoms include a dry cough, fever, headache, and dyspnea, and are often indistinguishable from those caused by other common bacterial and viral pathogens.

It has been observed that CAP caused by *M. pneumoniae* presents with highly variable symptoms. For example, in children over 3 years of age, it is often associated with a significantly longer duration of fever (>2 days) compared to other causes of CAP. The combination of these symptoms, along with a lack of response to empirical treatment with amoxicillin, leads clinicians to consider *M. pneumoniae* as a potential etiologic agent [4].

Since October 2023, the World Health Organization (WHO) [6] has been monitoring Chinese surveillance data concerning a rise in respiratory diseases in children in the northern regions of China. Reports of primary childhood pneumonia due to *M. pneumoniae* have also emerged from Europe and other countries, including France [7], Ireland, the Netherlands, and South Korea. This has increased the attention on the diagnostics and surveillance of *M. pneumoniae*.

The optimal specimen to collect for diagnosis is sputum, but throat or nasal swabs, bronchoalveolar washings, and, in instances of more severe infections, cerebrospinal fluid may also be utilized [3].

*M. pneumoniae* infection can be diagnosed using culture, serology, or molecular-based techniques, each with relative advantages and disadvantages [8].

The culture-based approach is time-consuming and has low sensitivity, as *M. pneumoniae* has stringent growth requirements and exhibits a very slow growth rate—it can take from 1 to 3 weeks to obtain a positive result. Moreover, growth on agar requires incubation in an atmosphere enriched with 5% CO_2_ and observation with a stereomicroscope to identify the small ‘fried-egg’ shaped colonies. Given these constraints, culture-based methods are rarely used and are mostly relegated to research purposes. The introduction of automated enzyme immunoassays for *M. pneumoniae* serology, replacing traditional complement fixation techniques, has enhanced testing efficiency and reduced turnaround times. The detection of serum IgM is indicative of acute or recent infection; however, serological tests exhibit low specificity and sensitivity due to cross-reactions with other microorganisms from the same genus. Although the combined IgM and IgA test may enhance sensitivity in the serological diagnosis of acute *M. pneumoniae* infection, further evaluations are needed to confirm the diagnosis [1]. When conducted concurrently with molecular tests, such as nucleic acid amplification tests (NAATs), serology can help in the identification of active infections. Thus, according to current clinical guidelines, the diagnosis of *M. pneumoniae* infections should be supported by both molecular and serological testing [9]. Presently, NAATs stand as the main diagnostic methods, as they offer high sensitivity and specificity and provide timely results for treatment decisions. A variety of molecular assays targeting distinct genes of *M. pneumoniae* are available, and some of them are included in commercial syndromic panels for detecting respiratory pathogens [8]. In particular, NAATs are advantageous for the early detection of the infection, but they cannot discriminate between an active infection and a carrier state [1,4,8].

Considering the increasing interest in *M. pneumoniae* diagnostics, due to its growing circulation and detection in pediatric patients, the aim of this study is to validate a molecular assay for the automated detection of *M. pneumoniae* by translating the in-house real-time PCR assay employed in the diagnostic routine to the fully automated Panther Fusion^®^ System (Hologic). The Panther Fusion^®^ System has been engineered for emergency scenarios, allowing a high-throughput analysis with continuous sample loading. We seek to ascertain the reliability of this system as a rapid diagnostic and high-throughput tool for specifically identifying *M. pneumoniae* infections by evaluating its sensitivity, specificity, and overall performance.

## 2. Materials and Methods

### 2.1. Specimen Enrollment

A retrospective study was conducted on 62 upper respiratory tract samples obtained from symptomatic children presenting to the Pediatric Emergency Unit of Padua University Hospital in 2023. The study cohort comprised patients with a mean age of 8.5 years (median: 8 years; range: 1–15 years). Of these, 32 tested positive for *M. pneumoniae* using an in-house real-time PCR method, which is currently employed for the molecular diagnosis of *M. pneumoniae*. This method was previously validated and certified according to the guidelines reported in references [10,11,12].

Two reference samples with distinct concentrations of *M. pneumoniae* were employed as controls: QC1 (approximately 150 genomic copies/mL) and QC2 (approximately 5000 genomic copies/mL). These QC samples were prepared by spiking *M. pneumoniae*-negative upper respiratory tract materials from patients with known concentrations of *M. pneumoniae*-positive samples.

Additionally, QCMD (Quality Control for Molecular Diagnostics) samples were used to further validate the new diagnostic approach. These external quality control samples are designed to assess the performance of molecular assays and to ensure consistency across different platforms.

### 2.2. In-House Real-Time PCR

For routine diagnostic testing of clinical samples, the procedure involves two main steps: (1) DNA extraction, performed using 0.2 mL of sample on the MagNA Pure 96 extraction platform (Roche, Milan, Italy) and (2) real-time PCR on the QuantStudio^™^ 5 System, following reported as-QS5 (Applied Biosystem by Thermo Fisher Scientific, Waltham, MA, USA).

The reaction mix consists of the following: 10 μL of extracted nucleic acids, 3 μL of the primer-probe mix, 15 μL of TaqMan™ Universal PCR Master Mix (Thermo Fisher Scientific, Milan, Italy), and 2 μL of H_2_O. Specifically, the primer-probe mix is composed of 30 μL of forward primer [100 μM], 30 μL of reverse primer [100 μM], 10 μL of probe [100 μM], and 230 μL of H_2_O. The thermal profile used is as follows: 2 min at 50 °C and 10 min at 95 °C, followed by 45 cycles of 15 s at 95 °C and 1 min at 60 °C. The primers and probe used in this study (Table 1) were previously described [13,14]. These primers and probe specifically target the RepMp1 gene, a repetitive sequence found within the *M. pneumoniae* genome, providing a theoretical advantage of improving sensitivity for detection [8,15]. Real-time PCR of the β-globin gene was conducted as an internal control for DNA extraction [16].

### 2.3. Generation and Quantification of the Positive Control

The amplicon generated by the primers reported in Table 1 was cloned into the plasmid pJeM1 (#135088, Addgene, Watertown, MA, USA). This plasmid was amplified in *E. coli*, purified, and quantified to be used as a positive control in Real-Time PCR assays, as well as for the optimization purposes of this study.

For plasmid quantification, a droplet digital PCR (ddPCR) system (Bio-Rad, Milan, Italy) was adopted. The reaction mixture described in Section 2.2 for the detection of *M. pneumoniae* was prepared, followed by the addition of the Bio-Rad ddPCR supermix. Next, oil was added into the designated wells of the cartridge, and using the QX100 Droplet Generator (Bio-Rad, Milan, Italy), an emulsion of approximately 20,000 droplets was generated. Each droplet, from a probabilistic standpoint, contained a single target along with the necessary amount of reaction mix to allow amplification. At this stage of the process, the droplets were transferred to a 96-well plate, and then the amplification cycle was performed. Upon completion of the PCR reaction, the samples were placed into the QX200 Droplet Reader (Bio-Rad, Milan, Italy), which analyzes the contents of each droplet individually to determine how many were positive or negative for the target. Finally, the QuantaSoft (version 1.7) software statistically processes the data, yielding the number of copies/μL of the target initially present in the sample.

### 2.4. Panther Fusion^®^ System

The analysis of the samples was performed on the Hologic Panther Fusion^®^ System (Hologic, Rome, Italy) using the Open Access functionality. This fully automated system is capable of extracting, amplifying, and detecting the patient’s material in a single run.

A 0.5 mL aliquot of the sample swab was added to the Multi Specimen Swab tube for testing on the Panther Fusion^®^ System. The FCR-S/FER-S reagent kit, containing Hologic’s Internal Control DNA, was used for nucleic acid extraction. The sample was eluted in 50 µL. Five µL of the eluted sample served as a template for the amplification reaction using the Panther Fusion^®^ open access RNA/DNA Enzyme Cartridge. Primers and probe concentrations were identical to those used in the in-house method, while the MgCl_2_ concentration was optimized through preliminary testing, resulting in a final concentration of 4 mM. The PCR cycling parameters on Hologic’s Panther Fusion^®^ System were set as follows: 95 °C holding stage for 2 min, followed by 43 cycles of 95 °C for 8 s, and 60 °C for 33 s.

### 2.5. Performance Experiments

Analytical performance was tested by evaluating the sensitivity, 95% limit of detection (LoD), precision and uncertainty, linearity, and correlation between the two platforms.

The 95% LoD was determined through serial dilutions of a clinical sample positive for *M. pneumoniae*, previously quantified by ddPCR. Twenty-four replicates were analyzed for each dilution. Precision and uncertainty of the assay were evaluated by testing the repeatability of results obtained with the QC1 and QC2 samples. QC1 and QC2 samples were analyzed in duplicate, with one or two series per day for two analytical sessions employing different operators over twenty days. Linearity was assessed using dilutions of a plasmid DNA containing the *M. pneumoniae* target sequence.

### 2.6. Statistical Analyses

Data were presented as numbers, percentages, or as coefficients of variation (CV). Linear regression analysis was conducted to determine the correlation coefficients (R2) using Statgraphics 19 software (Centurion). The statistical analysis was performed using the Shapiro–Wilk test for normality and the ANOVA tests.

## 3. Results

In order to validate a protocol for the automated diagnosis of *M. pneumoniae*, we transferred the in-house real-time PCR method currently used for the diagnostics to a fully automated Panther Fusion^®^ System. This system encompasses all of the analytical steps, from nucleic acid extraction to the generation of results after real-time PCR analyses.

The validation process included the following: (1) comparative assessment between the in-house methodology and the automatic platform for precision, uncertainty, linearity, and analytical sensitivity, and (2) amplification correlation tests conducted on both systems.

The specificity of primers and probe sequences was not further investigated in this study, as it was previously performed in the original publications [13,14].

The validation results are presented below.

### 3.1. Determination of the Limit of Detection (LoD)

The analytical limit of detection (LoD) for the QS5 and Panther Fusion^®^ platforms was determined by focusing exclusively on the real-time PCR amplification phase, using a positive sample for *M. pneumoniae*. The sample concentration was quantified using droplet digital PCR (ddPCR). Following quantification, the sample was amplified in 24 replicate reactions; the outcomes for the QS5 System are presented in Table 2.

The determination of the LoD was conducted using Statgraphics 19 software, yielding a result of 0.54 copies/reaction with a 95% probability. Considering the dilution factor, the LoD is established at 25 copies/mL (lower coefficient limit: 15 copies/mL and upper coefficient limit: 65 copies/mL).

For the Panther Fusion^®^ System, the sample was amplified in 20 replicate reactions. The results are presented in Table 3.

The determination of the LoD was performed using Statgraphics 19 software, yielding a result of 0.75 copies/reaction with a 95% probability. Considering the dilution factor, the LoD is established at 46 copies/mL (lower coefficient limit: 33 copies/mL and upper coefficient limit: 85 copies/mL).

### 3.2. Precision and Uncertainty

The assessment of precision was performed according to the recommendations of the CLSI MM6-A guidelines. The degree of agreement among repeated tests on the same sample was estimated under two conditions: the replication of the test on the same day (within-day precision) and the replication of the test on different days (between-day precision). QC1 and QC2 samples were analyzed in duplicate, one or two series per day for two analytical sessions, employing different operators per twenty days. The results are shown in Table 4.

The ANOVA test did not reveal any statistical differences between the QC1 and QC2 values obtained with the QS5 and Panther Fusion^®^ Systems.

### 3.3. Linearity

Linearity was assessed using plasmid DNA samples containing a diluted *M. pneumoniae* target sequence; the calculation was performed according to the recommendations of the CLSI EP6 guidelines. The analysis reveals that both the amplification systems exhibit excellent linearity for *M. pneumoniae* DNA concentrations ranging from 16 to 1.6 × 10^8^ copies/reaction for QS5 (R^2^ = 0.999) and 16 to 1.6 × 10^7^ copies/reaction for Panther Fusion (R^2^ = 0.998).

### 3.4. Correlation Test Between QS5 and Panther Fusion^®^ System

**Figure 1:**
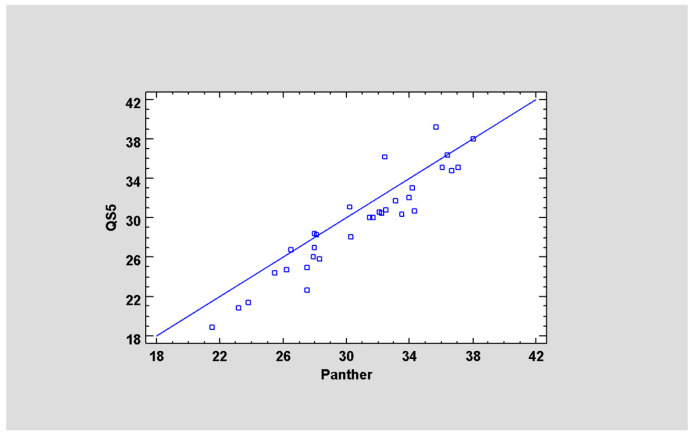
Linear correlation between the Ct values of the QS5 System and Panther Fusion^®^ System.

To verify the correlation between the two systems, a total of 32 positive samples for *M. pneumoniae* were used, and the results obtained were analyzed using the Statgraphics 19 software. All samples tested positive on both systems (100% agreement). The equation of the fitted model is as follows:

Panther Ct = 6.91 + 0.81 × QS5 Ct(1)

with a correlation coefficient of 0.94. The ANOVA analysis revealed a *p*-value lower than 0.05; the R^2^ statistic is 88.5% (Figure 1).

Thirty negative samples were also analyzed on both the platforms, resulting in a 100% agreement.

In addition to these tests, the laboratory participates in the QCMD interlaboratory comparisons. In 2024, the received panel was analyzed using both QS5 and Panther Fusion^®^ systems, revealing a remarkable concordance between the two platforms (Table 5).

Overall, the results demonstrated that the Panther Fusion^®^ System exhibited 100% positive predictive value and 100% negative predictive value when compared to the QS5 System for the detection of *M. pneumoniae*.

### 3.5. Diagnostic Use of the M. pneumoniae Test on the Panther Fusion^®^ System

**Figure 2:**
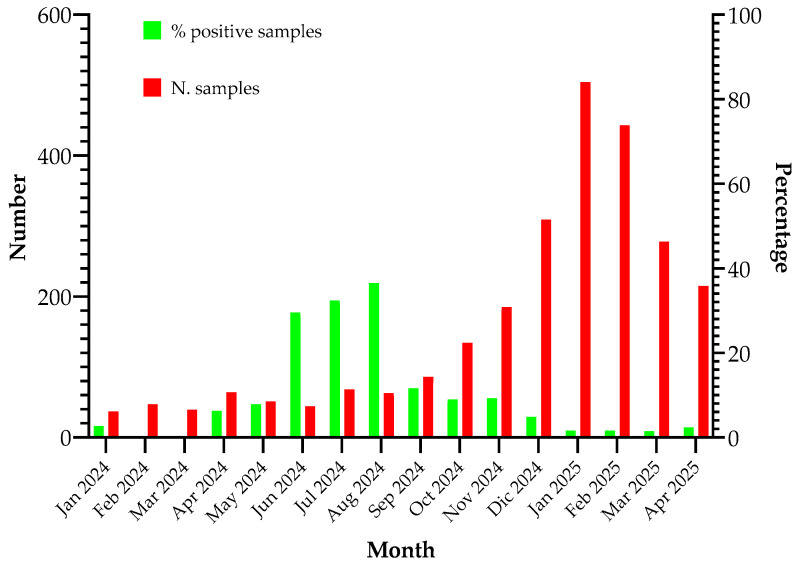
Monthly distribution of clinical samples analyzed using the Panther Fusion^®^ System. The percentage of positivity was also reported.

Starting from January 2024, the Panther Fusion^®^ platform was implemented for the diagnosis of *M. pneumoniae* infections in respiratory samples, alongside the detection of influenza viruses, SARS-CoV-2, and RSV using the commercially available Panther Fusion^®^ SARS-CoV-2/Flu A/B RSV assay (Hologic) [17]. A total of 2567 respiratory samples were analyzed, and the percentage of samples positive for *M. pneumoniae* is illustrated in Figure 2.

## 4. Discussion

*Mycoplasma pneumoniae* is a significant respiratory pathogen, accounting for up to 40% of cases of community-acquired pneumonia. Its impact on public health is considerable, affecting both children and adults globally. The microorganism’s ability to cause extrapulmonary complications, coupled with its potential for antibiotic resistance, makes it a growing concern in the medical community [2,3]. The recent *M. pneumoniae* epidemic has underscored the urgent need for enhanced diagnostic capabilities. This outbreak, which has affected numerous countries in Asia, has exerted significant stress on healthcare systems. The development of an automated diagnostic system could significantly improve the detection of *M. pneumoniae*, resulting in (i) more timely and accurate diagnoses; (ii) improved patient outcomes; and (iii) enhanced antibiotic stewardship [3].

The validation of the fully automated *M. pneumoniae* detection procedure was conducted throughout 2023. The analysis was performed on specimens obtained from the upper respiratory tract, which were then processed using two distinct methodologies. The first approach involved nucleic acid extraction followed by amplification with real-time PCR on the QuantStudio™ 5 (QS5) PCR Real-Time System (the in-house methodology used in routine diagnostics). The second method employed the Panther Fusion^®^ System (Hologic), a fully automated process suitable for emergency scenarios. This system offers several advantages, including continuous sample loading capability, faster processing compared to traditional methods, user-friendliness, the reduction of turnaround time and costs, and a significantly reduced risk of human error. Both methods utilize identical primers and probe sequences, enabling the amplification of the RepMp1 region of *M. pneumoniae*.

Sensitivity and limit of detection (LoD). The LoD of the Panther Fusion^®^ System was determined to be 46 copies/mL, which aligns with the high sensitivity required for detecting low microbial loads in clinical samples. Although the QS5 System exhibited a marginally lower LoD (25 copies/mL), this difference between the LoDs of the tests performed on the two platforms is unlikely to significantly impact the diagnostic efficacy of the Panther Fusion^®^ System [3,18]. Additionally, the LoD achieved with our real-time PCR assay on the Panther Fusion^®^ System performs better (46 copies/mL vs. 111 copies/mL) than that obtained in a recently published test optimized for the same platform and designed to amplify a different region of the *M. pneumoniae* genome [19]. Indeed, this system demonstrated reliable performance in detecting *M. pneumoniae* at clinically relevant concentrations, ensuring its effective utilization for diagnosing infections in clinical settings. The discrepancy in LoD between the two platforms can be attributed to differences in the reagents (type of polymerase and buffer and ion concentrations) employed for the DNA amplification and in the performance of the thermocycler.

Precision and uncertainty. The precision of the Panther Fusion^®^ System was assessed by repeating the test under different conditions, including within-day and between-day precision. Although the within- and between-day variability in the fully automated Panther Fusion^®^ System is higher than in the QS5 PCR Real-Time, in particular for the QC1 sample, the results demonstrated a remarkable improvement in Ct values obtained with the Panther Fusion^®^ System. This discrepancy between the two platforms could be attributed to differences in sample preparation methodologies. In the QS5 protocol, the sample is used directly without dilution, whereas in the Panther Fusion^®^ System, the sample undergoes dilution in the extraction tube prior to processing, which can affect the precision of target detection at low concentrations. However, this did not affect the general efficiency of the Panther Fusion^®^ System, as the overall results show 3 Ct less than the QS5 PCR Real-Time. This observation correlates with the extended uncertainty values that are lower for the QC2 in the Panther Fusion^®^ System while the opposite was observed for QC1. Additionally, our data are comparable to those recently reported for a similar assay optimized on the Panther Fusion^®^ System [19].

Linearity. The agreement between the linearity of the systems was excellent, with R^2^ values approaching 1.0. Although the QS5 system presented a broader dynamic range—up to 1.6 × 10^8^ copies/reaction compared to 1.6 × 10^7^ for the Panther Fusion^®^ System—linearity was confirmed for both platforms. Both systems exhibited remarkable performance across the entire concentration range, which is crucial for accurate detection in diagnostic settings where varying levels of *M. pneumoniae* need to be detected.

Correlation between Panther Fusion^®^ System and QS5 PCR Real-Time. A correlation analysis was carried out to assess the relationship between the Panther Fusion^®^ System and QS5 PCR Real-Time. The correlation coefficient of 0.94, along with the R^2^ statistic of 88.5%, suggests a robust concordance between the two systems. This assertion is further substantiated by the ANOVA analysis, which showed statistical significance (*p*-value < 0.05). The fitted model equation indicates a nearly linear relationship, suggesting that both systems provide comparable results for detecting *M. pneumoniae*. The observed agreement between the two platforms suggests that the Panther Fusion^®^ System may serve as a viable tool in clinical practice. In addition, a 100% concordance in results obtained with the two platforms further supports the suitability of the Panther Fusion^®^ System in diagnostic routine and emergency settings. Similar results were recently reported for a laboratory-developed test for *M. pneumoniae* comparing the Panther Fusion^®^ System with the Qiagen QIAsymphony platform [19].

Moreover, participation in the QCMD interlaboratory comparisons in 2024 provided additional validation of both systems. The panel analysis revealed consistent results across different laboratories, confirming that both the QS5 and Panther Fusion^®^ System are reliable for detecting *M. pneumoniae* in various settings. This consistency between laboratories is crucial to ensure that test results are not only accurate but also reproducible, regardless of the location of the analysis.

Diagnostic use of the *M. pneumoniae* test on the Panther Fusion^®^ System. The implementation of the Panther Fusion^®^ System facilitated the inclusion of *M. pneumoniae* diagnostics in the panel of respiratory pathogens investigated during the influenza season, resulting in a significant increase in the number of tests performed. The quantity of positive samples corroborates the typical seasonal distribution of *M. pneumoniae*, with peak incidence observed during summer and autumn, reaching up to 38% of positive samples in August.

In conclusion, the diagnostic tests performed on the Panther Fusion^®^ System demonstrated remarkable efficacy in detecting *M. pneumoniae*, exhibiting high sensitivity, accuracy, and precision, while showing substantial concordance with the QS5 results. In addition, the analytical performance is comparable with most commonly used diagnostic assays or similar in-house assays [3,19]. Given the global increase in *M. pneumoniae* infections, particularly among children, and the necessity for prompt treatment, the availability of reliable and efficient diagnostic systems is crucial for timely intervention with appropriate antibiotic therapy, thereby mitigating the risk of developing antibiotic-resistant strains [20]. Consequently, the Panther Fusion^®^ System emerges as a highly effective alternative to traditional in-house methodologies.

## Figures and Tables

**Table 1:** Oligonucleotides and probe used for the detection of *M. pneumoniae* [13,14].

Target	Primer	Sequence
	Forward	TCTTTACGCGTTACGTATTC
RepMp1	Reverse	AGTGTGGAATTCTCTGGCA
	Probe	(FAM)-TTCACTGGTATAACCGGTTTGTTAAG-(BHQ1)

**Table 2:** Analytical LoD of *M. pneumoniae* real-time PCR test on the QS5 platform using quantified plasmid control.

DNA Copies/Reaction	N° of Positive Samples (%)
8	24/24 (100%)
0.8	24/24 (100%)
0.4	18/24 (75%)
0.08	8/24 (33%)

**Table 3:** Analytical LoD of *M. pneumoniae* real-time PCR test on the Panther Fusion^®^ System using quantified plasmid control.

DNA Copies/Reaction	N° of Positive Samples (%)
6.10	20/20 (100%)
0.60	18/20 (90%)
0.3	12/20 (60%)
0.06	1/20 (5%)

**Table 4:** Summary of the results from the precision and uncertainty evaluation tests of *M. pneumoniae* DNA obtained with QS5 and Panther Fusion Systems.

	QS5		Panther Fusion	
	QC1	QC2	QC1	QC2
Average concentration (copies/mL)	150	5250	150	5250
Average concentration (copies/reaction)	3	105	2.23	78
Average Ct	36.4	31.5	33.7	29.8
Ct standard deviation of repeatability (within-day)	0.316	0.2	1.01	0.23
Ct standard deviation of repeatability (between-day)	1.31	0.72	2.01	0.41
Ct extended uncertainty#break# (95% confidence level)	2.74	1.51	4.2	0.85

**Table 5:** Comparison of the results of QCMD samples analyzed on the QS5 and Panther Fusion^®^ platforms.

QCMD Sample	QS5 (Ct)	Panther Fusion (Ct)
QCMD-1	29.1	28.3
QCMD-2	Neg	Neg
QCMD-3	35.9	32.1
QCMD-4	33.3	31.3
QCMD-5	34	32.9

## Data Availability

The original contributions presented in this study are included in the article. Further inquiries can be directed to the corresponding authors.
